# Colonic metastasis from infiltrating ductal breast carcinoma in a male patient: A case report

**DOI:** 10.1016/j.ijscr.2018.11.019

**Published:** 2018-11-16

**Authors:** Ashley Jones, Madison R. Kocher, Ashlee Justice, Fernando Navarro

**Affiliations:** a Department of Surgery, Palmetto Healthcare System, University of South Carolina Medical School, 2 Medical Park Road, Columbia, SC 29203, United States; b Department of Radiology, Medical University of South Carolina, 171 Ashley Avenue, Charleston, SC 29425, United States

**Keywords:** CT, computed tomography, EGD, esophagogastroduodenoscopy, ER, estrogen-receptor, FDG, fluorodeoxyglucose, GI, gastrointestinal, H&E, hematoxylin and eosin, PET, position emission tomography, PET-CT, positron emission tomography-computed tomography, PR, progesterone-receptor, Metastatic breast carcinoma, Breast carcinoma, Colonic metastases, Male breast carcinoma, Ductal carcinoma, Case report

## Abstract

**Introduction:**

A colonic metastasis from infiltrating breast ductal carcinoma is a rare phenomenon in the literature, especially in a male.

**Presentation of case:**

We present a rare case of a 55-year-old male with a past medical history of breast cancer who presented with signs and symptoms of appendicitis. A computed tomography (CT) scan revealed acute appendicitis in addition to a 2.3 cm cecal mass that correlated with a hypermetabolic region on positron emission testing (PET) the previous year. Analysis of a previously biopsied axillary lymph node demonstrated infiltrating ductal carcinoma. After an appendectomy and a right hemicolectomy were performed, pathologic analysis of the specimen revealed metastatic ductal carcinoma to the cecum.

**Discussion:**

Gastrointestinal metastases of breast carcinoma are rare with colonic metastases occurring in approximately 3% of these cases. At the time of diagnosis of these colonic metastases, the disease is often times multifocal in the gastrointestinal tract. Solitary gastrointestinal metastases are less common than both secondary primaries and benign processes. Biopsies obtained during colonoscopy are often non-diagnostic, mandating surgical excision and pathologic examination.

**Conclusion:**

Although colonic metastases from a primary breast ductal carcinoma are rare, a low level of suspicion must be maintained in a patient with such a history presenting with abdominal symptoms.

## Highlights

• Breast carcinoma gastrointestinal metastases occur in approximately 3% of cases.
• Metastatic gastrointestinal breast carcinoma is a rare phenomenon in men.
• Lobular carcinoma gastrointestinal metastases are more common than ductal.
• Metastatic breast carcinoma leaves the mucosal layer intact.
• Surgical resection may be necessary for diagnosis.

## 1 Introduction

This work is reported in line with the Surgical Case Report Guidelines (SCARE) criteria [1]. Breast carcinoma can commonly metastasize to the liver, lung, and bones, however, lower gastrointestinal tract and colonic metastases are rare phenomena [2]. When it occurs, multifocal disease and diffuse intraabdominal dissemination as often already occurred [3]. Here, we report a rare case of a solitary, colonic metastasis of breast carcinoma in a male patient followed by a thorough review of the literature.

## 2 Case presentation

The patient was a 55-year-old African American male with a significant past medical history of known breast cancer, who presented to an academic teaching hospital in February of 2018 after a surveillance computed tomography (CT) scan of his chest, abdomen, and pelvis showed incidental acute appendicitis. The patient was asymptomatic on his original presentation but subsequently developed nausea and vomiting along with right lower quadrant abdominal pain in the following days resulting in admission to the general surgery service for treatment of appendicitis. The patient did not have any contributory family, drug, or psychosocial history.

**Fig. 1:**
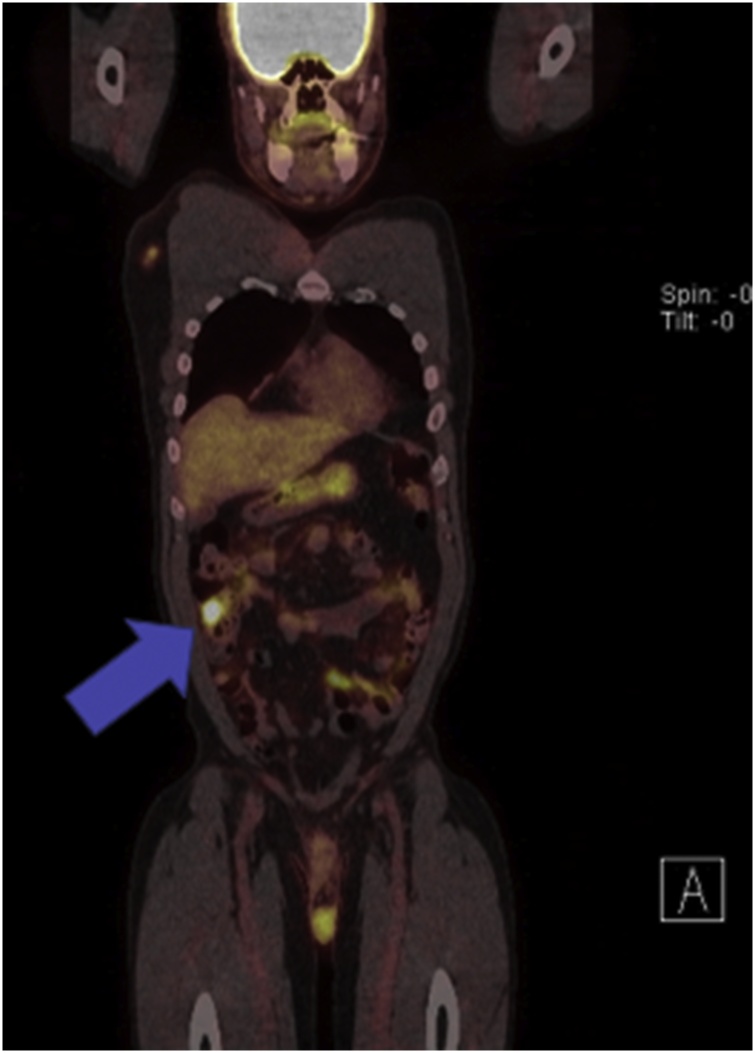
PET-CT scan of chest, abdomen, and pelvis. Coronal reformatted image obtained 90 min after IV administration of 14.0 mCi of F18-FDG demonstrating a solitary focus of intense activity noted within the cecum with a maximum standardized uptake value of 9.6 (denoted by the blue arrow). At the time, this was suspicious for a colonic primary tumor.

**Fig. 2:**
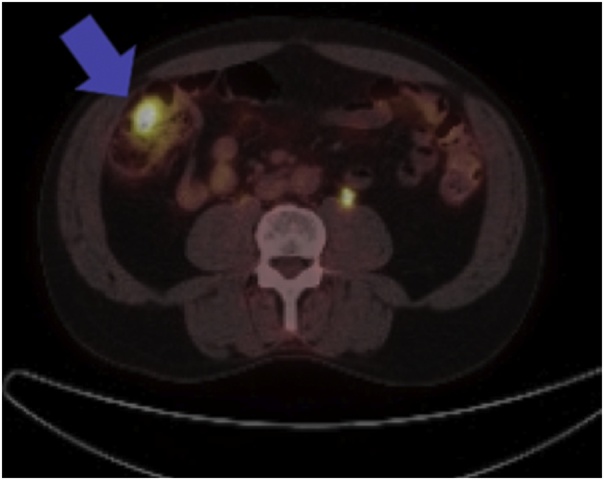
PET-CT scan of the chest, abdomen, and pelvis. Axial slice of the same PET-CT scan demonstrating the same hypermetabolic focus in the cecum (demonstrated by the blue arrow).

On chart review, the patient had an extensive past oncological history dating back to 2014 after resection of an enlarging, exophytic, ulcerating chest mass on the right side just lateral to the midline. Pathology from the wide local excision of this mass demonstrated estrogen receptor (ER) positive, progesterone receptor (PR) positive, and HER2/neu negative metastatic adenocarcinoma with an unknown primary source at the time. He underwent esophagogastroduodenoscopy (EGD) and colonoscopy to rule out a primary gastrointestinal malignancy, however, both were normal. He was followed closely by medical oncology and treated with tamoxifen for hormone therapy. Due to an enlarged anterior mediastinal wall lymph node discovered on routine CT surveillance in May of 2017, the patient underwent CT-guided biopsy and positron emission tomography (PET). The biopsy was negative for malignancy, however, the PET demonstrated abnormal, hypermetabolic activity within a retrosternal nodule, intense activity in a mixed focus in the manubrium, and several hypermetabolic nodes within the mediastinum. It also showed an abnormal hypermetabolic focus in the cecum suspicious for a colon primary (Fig. 1, Fig. 2). An attempt was made to complete a colonoscopy at the time, but the gastroenterologist could not advance the colonoscope past the transverse colon due to technical reasons. Medical oncology elected to continue close surveillance.

In January 2018, the patient was noted to have right axillary lymphadenopathy and an ultrasound-guided biopsy of a 1.5 cm right axillary lymph node was performed. Pathology results showed an infiltrating, moderately-differentiated ductal carcinoma. This specimen was also ER and PR positive as well as HER2/neu negative.

**Fig. 3:**
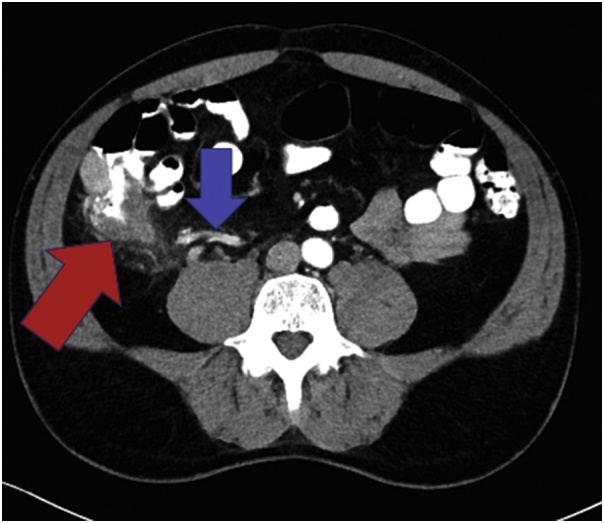
Abdominal CT image at admission with IV and oral contrast. Multiple contiguous axial images of the abdomen and pelvis were obtained following the administration of intravenous and oral contrast. The appendix is dilated to 1.8 cm with significant periappendiceal stranding and appendiceal wall thickening (blue arrow). Also in the right lower quadrant, either adjacent to or within the wall of the cecum, is a bowel mass measuring 2.3 × 1.9 × 2.3 cm, concerning for a malignancy (demonstrated by the red arrow).

A CT scan of the patient’s chest, abdomen, and pelvis was completed on February 15, 2018 for further monitoring. At this time, the patient was incidentally found to have a dilated appendix with no wall thickening or periappendiceal stranding. He did not have a leukocytosis and clinically he did not have any fever or any symptoms including pain, nausea, or vomiting at this original presentation. However, he returned two days later with nausea, vomiting, and right lower quadrant pain consistent with appendicitis. On a repeat CT of the abdomen and pelvis with IV and oral contrast, the appendix was dilated to 1.8 cm with appendiceal wall thickening and periappendiceal stranding. In addition, a 2.3 × 1.9 × 2.3 cm bowel mass was identified in the right lower quadrant either adjacent to or within the wall of the cecum that correlated with the previous PET-CT scan performed in May 2017 (Fig. 3). He was therefore admitted and treated with IV antibiotics for acute appendicitis with plans for appendectomy during his hospitalization along with further workup of the cecal mass.

Because of his oncological history, a colonoscopy was performed on hospital day one to evaluate the cecum to rule out metachronous colon tumors given his CT imaging findings and previous PET-CT. The colonoscope was advanced to the cecum without issue. Cecal inflammation was noted and several biopsies were taken of this area, however, no discernable tumors were noted. Pathology returned with no significant pathologic changes to the biopsied tissue.

The following day after receiving intravenous antibiotics and fluid resuscitation, the patient underwent laparoscopic appendectomy. The procedure was converted to open due to the extensive inflammatory process in the right lower quadrant and retrocecal position of the appendix. The appendix was divided from the cecum using a GIA stapler and the appendiceal vessels were suture ligated and divided with electrocautery. The specimen was removed from the field and sent to pathology for evaluation. Prior to abdominal closure, the colon was run from the terminal ileum to the rectum and a large walnut-sized mass was discovered in the cecum proximal to the area of the appendiceal orifice. It was hard, mobile, and suspicious for a primary colon lesion. A right hemicolectomy was then performed including adequate mesenteric lymph node resection. The operation was performed by a general surgeon and a chief resident. The patient tolerated the procedure without complications and was returned to the surgical floor postoperatively. His post-operative course was unremarkable, and he was discharged five days after the procedure.

**Fig. 4:**
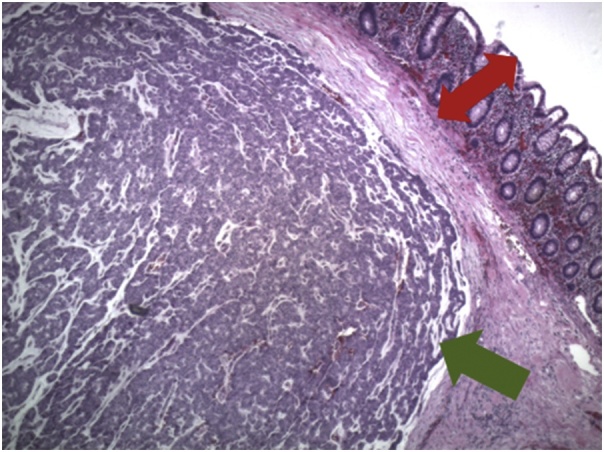
Hematoxylin and eosin (H&E) stain at 40x magnification demonstrating the tumor from the colectomy specimen (green arrow) within the submucosa with an overlying, benign colonic mucosa (red arrow).

**Fig. 5:**
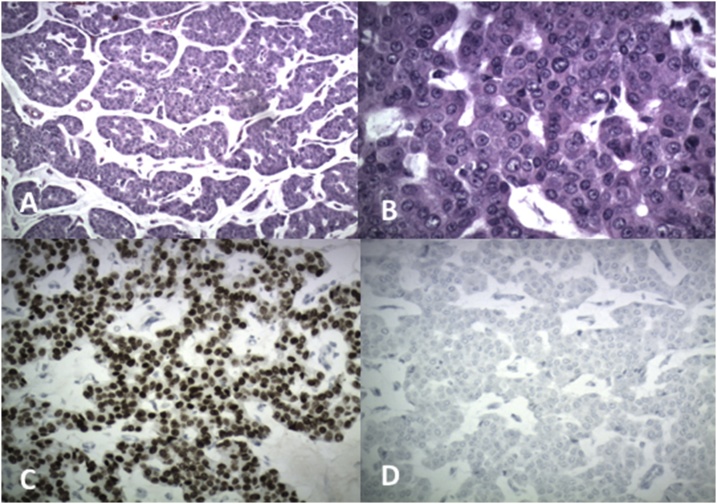
Histological examination. The following findings demonstrate invasive ductal breast carcinoma. A. Nests of tumor cells with basophilic mucin production (H&E of colon tumor, 100x magnification). B. Tumor composed of cords and nests of epithelial cells with eosinophilic cytoplasm and prominent nucleoli (H&E stain of colon tumor, 400× magnification). C. Positive nuclear staining for Gata3 (Gata3 immunohistochemical stain, 200× magnification). D. Negative for CDX2 (CDX2 immunohistochemical stain, 200× magnification).

Pathology from the surgical specimen returned shortly after the surgery. The appendix showed findings suggestive of acute appendicitis with organizing periappendicitis. The right colon specimen returned as metastatic breast carcinoma involving cecal submucosa clear of margins with overlying benign colonic mucosa (Fig. 4). Twenty lymph nodes were harvested and were all negative for disease. The specimen stained strongly and diffusely for GATA3 which was consistent with metastatic breast carcinoma and negative for CDX2 (Fig. 5A–D).

After discharge, the patient had close surveillance with medical oncology. He was transitioned to anastrazole secondary to the ER/PR + receptor status of his tumor and its refractory response to tamoxifen. Prior to his first outpatient appointment in April 2018, he complained of worsening skeletal pain at multiple sites. Bone scan showed left posterior rib and sternal involvement for which zoledronic acid was initiated. He also underwent CT scan of his chest, abdomen, and pelvis to monitor progression of his metastatic disease which showed no significant changes. During his appointment in June 2018, he was noted to have an enlarging left lateral neck mass. Ultrasound-guided biopsy of this mass returned positive for metastatic carcinoma consistent with breast origin. No significant changes to his treatment regimen have occurred since this time.

## 3 Discussion

Breast carcinoma commonly results in widespread dissemination that can involve many organs. While gastrointestinal metastases of breast carcinoma are an uncommon phenomenon occurring in approximately 4–18% of metastatic breast cancer patients, colonic metastases are extremely rare and occur in approximately 3% of cases [2,[4], [5], [6], [7]]. When it occurs, the rectum and the rectosigmoid region are most commonly affected and multifocal disease in the gastrointestinal system is often present [3]. Gastrointestinal metastases in general are rarely diagnosed antemortem but can be identified on autopsy [2]. In our presented case, the isolated gastrointestinal metastasis was localized antemortem in the ascending colon of a male patient. Even in patients with a history of breast cancer, it has been shown that solitary gastrointestinal metastases are less common than both secondary primaries and benign processes [8]. Table 1 describes the published cases with their respective treatments and survival following the diagnosis of metastatic disease to the colon or intestines.

**Table 1:** Published cases with treatment regimens and survival after the diagnosis of metastatic disease.

Case	Patient description	Original diagnosis	Metastases	Treatment of metastases	Survival after metastatic diagnosis
Uygun et al. [2]	43 year old female	Ductal and lobular mixed type carcinoma	Bone metastases and colonic metastasis found 3 years later	6 cycles of chemotherapy (5-fluorouracil, Adriamycin, and cyclophosphadmide), followed by megestrol acetate	Alive 7 months after the diagnosis of colonic metastases
Dar et al. [3]	75-year-old female	Comedo-type intraductal carcinoma	Distal sigmoid colon metastasis found 6 years later	Rectosigmoidectomy	Not reported
Tsujimura et al. [4]	51-year-old female	Invasive lobular carcinoma of the breast	Ascending colon tumor found concurrently	Ileocecal resection and letrozole	Stable disease 9 months postoperatively
Klein and Sherlock [6]	68-year-old female	Infiltrating lobular carcinoma	Stomach, mesentery, and sigmoid colon	Estrogen and prednisone therapy in addition to surgical resection of metastases	1 week (although diffuse metastatic disease prior)
	77-year-old female	Carcinoma	Stomach and sigmoid	Radiotherapy and estrogen therapy	“discharged for long term care”
Gifaldi et al. [9]	86-year-old female	Infiltrating lobular carcinoma	Colon	Tamoxifen	Not reported
Clavien et al. [10]	49-year-old female	Infiltrating lobular and ductal carcinoma	Stomach and peritoneal carcinomatosis	None	4 months
	82-year-old female	Infiltrating lobular carcinoma	Rectum	Tamoxifen	Alive after 18 months
	62-year-old female	Infiltrating lobular and ductal carcinoma	Retroperitoneal and peritoneal carcinomatosis	Intraperitoneal 5-fluorouracil and tamoxifen	6 months
	52-year-old female	Infiltrating lobular carcinoma	Retroperitoneal and peritoneal carcinomatosis	Adnexectomy, chlorambucil, methotrexate, 5-fluorouracil, epirubicine, tamoxifine	Alive after 7 years
Koos and Field [12]	52-year-old female	Infiltrating lobular carcinoma	Colon	Total colectomy	10 months
Weisbert [13]	69-year-old female	Infiltrating lobular carcinoma	Colon and mesentery	Refused	2 years

Large bowel metastatic disease can have variable and nonspecific presentations ranging from vague abdominal pain to changes in bowel habits. Acute symptoms are rarely experienced and an insidious onset of inflammatory bowel disease symptoms is more likely [3,9]. Nonspecific radiographic features, rarity of occurrence, and variable presentation mandate histologic examination after biopsy or surgical excision to diagnose intestinal metastases [2]. In this presented case, the patient was found to have a hypermetabolic lesion on screening PET-CT prior to presenting with acute appendicitis symptoms. Because of the cecal location of the increased FDG uptake, colonoscopy was performed prior to operative intervention for precise operative planning and further characterization of the lesion prior to appendectomy.

In this case, histological examination revealed metastatic breast carcinoma in the right colon from an infiltrating ductal carcinoma primary tumor, previously metastatic to an axillary lymph node. However, in most published case reports, lobular carcinoma is the most common primary tumor reported to metastasize to the gastrointestinal tract. In a study performed by Clavien et al., extrahepatic gastrointestinal metastases of women treated for breast cancer were examined. Although the histology of the primary tumors was primarily invasive lobular mixed with invasive ductal carcinoma in half of the specimens, only invasive lobular carcinoma was identified in the gastrointestinal metastases [10]. In another study performed by Taal et al., nearly all metastatic lesions to the colorectal region occurred in lobular carcinoma breast cancer patients [5]. Several other published case reports demonstrate colonic metastases from a primary lobular carcinoma of the breast [7,11,12].

Metastatic breast carcinoma to the colon is histopathologically unique in that the tumor is able to invade the muscle layer while leaving the mucosal layer histologically intact [12,13]. Therefore, if an attempt at a biopsy during a colonoscopy is made, deep tissue sampling would be crucial. Although an attempt to biopsy the ascending colonic mass was made during the colonoscopy for our patient, only colonic mucosa with intact crypt architecture was identified at pathology. Deeper biopsies would have been required for accurate identification of metastatic breast carcinoma.

Survival is unfortunately very poor after the discovery of gastrointestinal metastases with few patients surviving beyond two years, however, successful multimodality treatment with hormonal therapy or chemotherapy alone or as adjuvant therapy to surgical excision has been demonstrated [2,5,10].

## 4 Conclusion

Solitary gastrointestinal – and especially colonic – metastases are very rare in patients with breast cancer. Because colonoscopic biopsies may only capture normal mucosa, surgical resection and histopathologic correlation to the primary tumor is mandatory for diagnosis. A low threshold of suspicion should be maintained, especially in patients with a history of breast cancer and gastrointestinal symptoms, regardless of gender.

## Conflicts of interest

None of the authors have any financial or personal relationships with other people or organizations that could inappropriately influence our work.

## Sources of funding

There were no sources of funding for our work.

## Ethical approval

As this work is a case report, ethical approval was not needed and our report was exempt from ethical approval at our institution.

## Consent

Written informed consent was obtained from the patient for publication of this case report and accompanying images. A copy of the written consent is available for review by the Editor-in-Chief of this journal upon request.

All efforts have been made to omit non-essential details and to maintain the patient’s anonymity.

## Author contributions

Ashley Jones, DO: Study concept, writing, editing.

Madison Kocher, MD: Writing, editing, figures, and figure legends.

Ashlee Justice, MD: Writing, editing.

Fernando Navarro, MD: Editing, supervision.

## Registration of research studies

As this is a case report and not a human studies, it is exempt from registering.

## Guarantor

Fernando Navarro, MD.

## Provenance and peer review

Not commissioned, externally peer reviewed.
